# Molecular, Immunological, and Biological Characterization of *Tityus serrulatus* Venom Hyaluronidase: New Insights into Its Role in Envenomation

**DOI:** 10.1371/journal.pntd.0002693

**Published:** 2014-02-13

**Authors:** Carolina Campolina Rebello Horta, Bárbara de Freitas Magalhães, Bárbara Bruna Ribeiro Oliveira-Mendes, Anderson Oliveira do Carmo, Clara Guerra Duarte, Liza Figueiredo Felicori, Ricardo Andrez Machado-de-Ávila, Carlos Chávez-Olórtegui, Evanguedes Kalapothakis

**Affiliations:** 1 Departamento de Biologia Geral, Instituto de Ciências Biológicas, Universidade Federal de Minas Gerais, Belo Horizonte, Minas Gerais, Brazil; 2 Programa de Pós-Graduação em Ciências Biológicas: Fisiologia e Farmacologia, Instituto de Ciências Biológicas, Universidade Federal de Minas Gerais, Belo Horizonte, Minas Gerais, Brazil; 3 Departamento de Bioquímica-Imunologia, Instituto de Ciências Biológicas, Universidade Federal de Minas Gerais, Belo Horizonte, Minas Gerais, Brazil; Institut de Recherche pour le Développement, Benin

## Abstract

**Background:**

Scorpionism is a public health problem in Brazil, and *Tityus serrulatus* (Ts) is primarily responsible for severe accidents. The main toxic components of Ts venom are low-molecular-weight neurotoxins; however, the venom also contains poorly characterized high-molecular-weight enzymes. Hyaluronidase is one such enzyme that has been poorly characterized.

**Methods and principal findings:**

We examined clones from a cDNA library of the Ts venom gland and described two novel isoforms of hyaluronidase, TsHyal-1 and TsHyal-2. The isoforms are 83% identical, and alignment of their predicted amino acid sequences with other hyaluronidases showed conserved residues between evolutionarily distant organisms. We performed gel filtration followed by reversed-phase chromatography to purify native hyaluronidase from Ts venom. Purified native Ts hyaluronidase was used to produce anti-hyaluronidase serum in rabbits. As little as 0.94 µl of anti-hyaluronidase serum neutralized 1 LD_50_ (13.2 µg) of Ts venom hyaluronidase activity *in vitro*. *In vivo* neutralization assays showed that 121.6 µl of anti-hyaluronidase serum inhibited mouse death 100%, whereas 60.8 µl and 15.2 µl of serum delayed mouse death. Inhibition of death was also achieved by using the hyaluronidase pharmacological inhibitor aristolochic acid. Addition of native Ts hyaluronidase (0.418 µg) to pre-neutralized Ts venom (13.2 µg venom+0.94 µl anti-hyaluronidase serum) reversed mouse survival. We used the SPOT method to map TsHyal-1 and TsHyal-2 epitopes. More peptides were recognized by anti-hyaluronidase serum in TsHyal-1 than in TsHyal-2. Epitopes common to both isoforms included active site residues.

**Conclusions:**

Hyaluronidase inhibition and immunoneutralization reduced the toxic effects of Ts venom. Our results have implications in scorpionism therapy and challenge the notion that only neurotoxins are important to the envenoming process.

## Author Summary

In Brazil, accidents with scorpion stings have been a serious public health problem, and *Tityus serrulatus* (Ts) is primarily responsible for severe accidents. Therefore, efforts have been made to understand the characteristics of the molecules present in scorpion venoms. These venoms are complex mixtures, in which neurotoxins are the main toxic components. Ts venom also contains enzymes, such as hyaluronidase, that have not been well characterized. In this study, we described for the first time two sequences of Ts hyaluronidase isoforms: TsHyal-1 and TsHyal-2. We purified native hyaluronidase from Ts venom and produced anti-hyaluronidase serum in rabbits. This serum neutralized hyaluronidase activity present in Ts venom. *In vivo* neutralization assays showed that anti-hyaluronidase serum inhibited and delayed mouse death after injection of a lethal dose (50% lethal dose, LD_50_) of Ts venom. This work confirms the influence of hyaluronidase in Ts venom lethality and paves the way for the development of new strategies for scorpionism therapy.

## Introduction

Accidents with scorpion stings have been a serious public health problem in Brazil for many decades. In 2007, the World Health Organization (WHO) officially established scorpion sting envenoming as a neglected public health issue in many parts of the world, especially developing countries [1]. In 2010, the Brazilian Academy of Sciences (ABC) recognized the accidents caused by venomous animals as neglected tropical diseases [2].

In Brazil, scorpion accidents have the highest incidence when compared to those caused by other venomous animals, including snakes [3], [4]. The yellow scorpion *Tityus serrulatus* (Ts) is the species primarily responsible for severe accidents because it is easily adapted to the urban environment [5], [6]. In 2012, the Brazilian Ministry of Health reported approximately 63,000 cases of scorpion stings, and some of them led to death [3]. The mortality rate from scorpion stings is approximately 1.77% among children up to 14 years old, but most accidents occur in adults [7], [8].

Treatment of moderate and severe scorpion stings consists of analgesics for pain and horse antiscorpionic or anti-arachnidic antivenom [9], [10]. Our research group has been studying alternative methods to produce antivenom sera without using crude venom. Advantages of eliminating the use of crude venom include a less-toxic immunization process and improved antivenom specificity [11]–[16]. Increasing antivenom specificity may enhance the effectiveness of scorpionism treatments.

To improve therapies, it is imperative to understand the functions of Ts venom components. Our group has recently profiled the transcriptome of the Ts venom gland and found new venom components [17]. The main toxic components of Ts venom are the well-described α and β-type toxins, which act on different sites of voltage-sensitive sodium channels [18]–[23]. Transcriptome analysis showed that sodium channel toxins represent 16% of the transcripts in Ts venom [17]. These neurotoxins disrupt the neuromuscular, cardiovascular, and respiratory systems and elicit a complex pattern of clinical signs and symptoms that can lead to death [9], [24]–[27]. Other important neurotoxins, which represent 22% of Ts venom transcripts [17], act on potassium channels [28]–[30]. Ts venom also contains bradykinin potentiating peptides, hypotensins, antimicrobial peptides, and anionic peptides [31]–[34], [17]. Metalloproteases, phospholipase-like and hyaluronidases are enzymes present in Ts venom as well [22], [35], [17], [36], however their importance in the envenomation process has been understudied.

To study the enzymatic activity of Ts venom, we focused on the enzyme hyaluronidase. Hyaluronidases are found in several animal venoms, such as hymenopteran, arachnid, and snake venoms [37]–[43]. The role of hyaluronidase in these venoms has been speculated throughout the years, but few evidences were shown so far. However, hyaluronidase is commonly known as a “spreading factor” because it hydrolyzes the hyaluronan (also known as hyaluronic acid, HA) of connective tissue, thus facilitating the invasion of venom toxins into the victim's organism and blood vessels, therefore acting as a catalyzer to the systemic envenomation [37], [44]. Furthermore, the degradation of HA produces small HA fragments, which are pro-inflammatory, pro-angiogenic and immunostimulating [45]. This immunostimulation induces physiological and pathological processes that promote faster systemic envenomation. Because hyaluronidase promotes the distribution of the toxic venom throughout the victim's tissues, it has been a focus of discussions regarding the efficacy of Ts antivenom treatment. Late administration of antivenom, in conjunction with the rapid spread of venom promoted by hyaluronidase, makes quick and effective neutralization of venom difficult to accomplish [46], [47], [22].

Although hyaluronidase activity was first described in Ts venom by Possani *et al.* [48], it was not isolated from Ts venom until 2001 by Pessini *et al.* [49]. Recently, Venancio *et al.* [36] showed increased hyaluronidase activity in Ts venom when compared to *T. stigmurus* venom. Furthermore, there is minimal sequence information available for Ts hyaluronidase. Until this study, the only available Ts sequence was a 34-amino acid sequence obtained by Edman degradation of the N-terminus (UniProtKB ID: P85841; submitted by Dr. Michael Richardson's group in 2008).

The aim of this study was to isolate, sequence, and molecularly and immunologically characterize Ts hyaluronidase obtained from cDNA clones, as well as to purify and characterize native hyaluronidase from Ts venom. We report the cDNA and predicted amino acid sequences of two Ts hyaluronidase isoforms. We also produced a rabbit serum against Ts native hyaluronidase, which recognized common epitopes to both isoforms, including residues from the active site. This anti-hyaluronidase serum neutralized 1 LD_50_ of Ts venom in mice and delayed time of death, thus indicating the importance of hyaluronidase in the envenomation process.

## Materials and Methods

### Drugs

Aristolochic acid was purchased from Sigma-Aldrich (St. Louis, MO, USA), dissolved in ethanol at 5 mg/ml, and stored at 4°C until needed. It was diluted in phosphate-buffered saline (PBS) when used in the pharmacological inhibition protocols. HA sodium salt from *Streptococcus equi* (Sigma-Aldrich) was dissolved in ultra-pure water at 2.5 mg/ml and stored at −20°C. Commercial hyaluronidase from bovine testes (Apsen, São Paulo, Brazil) was dissolved in ultra-pure water at 5 mg/ml and stored at −20°C.

### Scorpions and venom

Adult Ts scorpions were collected in the region of Belo Horizonte, Minas Gerais, Brazil, with a license from the Brazilian government for collection and maintenance of scorpions (IBAMA, Instituto Brasileiro do Meio Ambiente e dos Recursos Naturais Renováveis, protocol 31800). Crude venom was obtained by electrical stimulation of the telsons. Venom was diluted in ultra-pure water and centrifuged (16000× g, 10 min, 4°C) to remove insoluble materials. The supernatant was stored at −20°C. Protein concentration of the venom supernatant was measured as described by Lowry *et al.* [50].

### Ethics statement

The Ethics Committee of the Federal University of Minas Gerais (“Comitê de Ética no Uso de Animais”, CEUA) certifies that the procedures using animal in this work are in agreement of the Ethical Principals established by the Brazilian Council for the Control of Animal Experimentation (CONCEA). Protocol number 326/2012. Approved: March 14, 2013.

### Experimental animals

Female Swiss CF1 mice (18–22 g) from the Animal Care Facilities (CEBIO) at the Federal University of Minas Gerais (UFMG) were used. Adult female New Zealand white rabbits (2–2.5 kg) were obtained from the Animal Facilities Center of the School of Veterinary Medicine, UFMG. All animals had free access to water and food under controlled environmental conditions. Animal experiments were performed according to the Brazilian Council for Animal Care guidelines and were approved by the Ethics Committee (CEUA, protocol 326/2012) of UFMG.

### Hyaluronidase cDNA sequencing

Two hyaluronidase clones were isolated from a cDNA library of the Ts venom gland [51]. Screening was performed by transcriptome analysis of the venom gland [17]. DNA was resequenced with an ABI 3130 Genetic Analyzer using the BigDye Terminator Sequencing Kit v3.1 (Applied Biosystems, Foster City, CA, USA), standard M13 forward and reverse primers, and a gene-specific internal primer (5′-CGTGGCTATGGAATCAATCTACTG-3′). Resequencing was performed to obtain and confirm the complete sequences using both strands.

### Computer analyses

Blastx and Blastp were the local alignment search tools used to compare the Ts hyaluronidase sequence with other hyaluronidase sequences. Amino acid sequences were deduced and analyzed with the following software tools: ORF Finder from the National Center for Biotechnology Information (NCBI, http://www.ncbi.nlm.nih.gov/projects/gorf/), Translate Tool and Compute pI/Mw from the ExPASy World Wide Web server (http://www.expasy.org), and SignalP 4.1 Server (http://www.cbs.dtu.dk/services/SignalP/).

Ts hyaluronidase sequences were aligned with known hyaluronidase sequences from diverse organisms, including *Mesobuthus martensii* (GenBank: ACY69673; [52]), *Loxosceles intermedia* (GenBank: AGH25912; [42]), *Brachypelma vagans* (GenBank: AFS33217; [41]), *Polybia paulista* (GenBank: ADL 09135; [43]), *Apis mellifera* (UniProtKB/Swiss-Prot: Q08169; [53]), and *Homo sapiens* (UniProtKB/Swiss-Prot: Q12794; [54]). A multiple alignment was performed using the algorithm Clustal W in MEGA 5.1 software [55]. Prediction of N-glycosylation sites in Ts hyaluronidase sequences was performed using the online server GPP (Glycosylation Prediction Program) [56].

### Venom fractionation and purification of native Ts hyaluronidase

Purification of hyaluronidase from Ts venom was performed in two chromatographic steps. First, Ts venom was diluted to 225 mg/ml in 30% (v/v) acetic acid with ultra-pure water and fractionated by gel filtration chromatography. A column (100 cm×2.5 cm) of Sephadex G-50 Fine (GE Healthcare, Giles, UK) was equilibrated with 30% (v/v) acetic acid. Ts venom (450 mg) was applied to the column and 2 ml samples were collected at a flow rate of 6 ml/h of 30% (v/v) acetic acid solution. This chromatographic procedure was monitored by absorbance at 280 nm, and fractions corresponding to the same peak were pooled together and freeze-dried. Each pooled fraction was analyzed for protein concentration as described by Lowry *et al.* [50] and for hyaluronidase activity (described in the “Hyaluronidase activity: *in vitro* assay” section).

The active fraction obtained from the first step of gel filtration chromatography was diluted in solution A (0.1% (v/v) trifluoracetic acid [TFA; Sigma-Aldrich] in ultra-pure water), and 1 mg was applied to a reversed-phase C8 analytical column (300 Å, 5 µm, 4.6 mm 250 mm; Grace Vydac, OR, USA), previously equilibrated with solution A. The sample was eluted with a gradient of solution B (0.1% (v/v) TFA in acetonitrile [ACN; Merck, Darmstadt, Germany]) at a flow rate of 1 ml/min: 0–40% (v/v) B from 10 to 12 min, 40–75% (v/v) B from 12 to 37 min, 75–100% (v/v) B from 37 to 37.5 min, 100% (v/v) B from 37.5 to 45 min. This chromatographic procedure was monitored by absorbance at 214 and 280 nm. Fractions corresponding to the same peak were pooled together and freeze-dried. Reversed-phase chromatography (RPC) was performed with a Shimadzu Prominence HPLC (Shimadzu, Kyoto, Japan). Each pooled fraction was analyzed for hyaluronidase activity (described in the “Hyaluronidase activity: *in vitro* assay” section).

### Hyaluronidase activity: *In vitro* assay

Hyaluronidase activity was measured according to the turbidimetric method described by Pukrittayakamee *et al.* [57] with modifications. The assay mixture contained 12.5 µg of HA (Sigma-Aldrich), acetate buffer (0.2 M sodium acetate-acetic acid pH 6.0, 0.15 M NaCl), and test sample (or control sample) in a final volume of 250 µl. Commercial hyaluronidase from bovine testes (12.5 µg; Apsen) was used as positive control, and ultra-pure water was used as negative control. Assay mixtures were incubated for 15 min at 37°C, and reactions were stopped by adding 500 µl of stop solution, containing 2.5% (w/v) cetyltrimethylammonium bromide (CTAB) dissolved in 2% (w/v) NaOH.

Assays were monitored by absorbance at 400 nm against a blank of acetate buffer (250 µl) and stop solution (500 µl). Turbidity of the samples decreased proportionally to the enzymatic activity of hyaluronidase. Values were expressed as percentages of hyaluronidase activity relative to the negative (no enzyme addition, 0% activity) and positive (commercial enzyme addition, 100% activity) controls.

### Electrophoresis and mass spectrometry (MS) analysis

Ts venom (12.5 µg), a hyaluronidase active fraction from gel filtration chromatography (7 µg), and a hyaluronidase active fraction from RPC (2 µg) were submitted to electrophoresis under reducing condition using 15% (w/v) SDS-PAGE as described by Laemmli [58]. The gel was stained with Coomassie Blue (Sigma-Aldrich). The hyaluronidase active fraction from RPC was dissolved in a 1∶1 (v/v) ACN∶water solution containing 0.1% (v/v) TFA. Then, it was mixed 1∶1 (v/v) with 2,5-dihydroxybenzoic acid (DHB), and submitted to MS analysis. It was used matrix-assisted laser desorption ionization time-of-flight (MALDI-TOF) instrumentation with an Autoflex III (Bruker Daltonics, Billerica, MA, USA) operated in positive mode to determine the molecular weight of any compounds in the sample. Monoisotopic masses were obtained in positive linear mode. It was used mass ranges from 5 kDa to 20 kDa, and from 20 kDa to 100 kDa.

### Anti-hyaluronidase serum production

After collection of pre-immune sera, rabbits (n = 2) were injected subcutaneously at 4 locations with 50 µg of native Ts hyaluronidase emulsified in complete Freund's adjuvant (Sigma-Aldrich). Three consecutive boosters, each containing 100 µg of native Ts hyaluronidase, were emulsified in incomplete Freund's adjuvant (Sigma-Aldrich) and administered subcutaneously to each rabbit at 10-day intervals. One week after each booster, blood samples were drawn from the ear veins of the rabbits, and serum was extracted and stored at −20°C until needed. One week after the last injection, blood was drawn, and serum was extracted and titrated using an ELISA as described by Chavez-Olórtegui *et al.* [59]. Briefly, ELISA plates (BD, NJ, USA) were pre-coated with crude Ts venom (5 µg/ml) or native Ts hyaluronidase (5 µg/ml). Anti-hyaluronidase rabbit serum was titrated using dilutions ranging from 1∶100 to 1∶51,200. Absorbance was measured at 492 nm.

### *In vitro* neutralization assay

First, Ts venom toxicity was assayed in naive female Swiss CF1 mice [18–22 g] by subcutaneous injection. The number of dead animals was determined within 24 h, and the LD_50_ of Ts venom was estimated by the method of Karber [60]. The subcutaneous LD_50_ of the Ts venom used throughout this study was 13.2 µg per 20 g mouse. To establish the minimum volume of rabbit anti-hyaluronidase serum sufficient to neutralize the *in vitro* hyaluronidase activity of Ts venom, different volumes of anti-hyaluronidase serum (serially diluted by a factor of 1.5) were incubated with Ts venom (1 LD_50_ = 13.2 µg) for 1 h at 37°C before the turbidimetric assays were performed (described in the “Hyaluronidase activity: *in vitro* assay” section).

In another set of experiments, it was determined the minimum quantity of native Ts hyaluronidase required to recover 100% of the enzymatic activity in a sample of neutralized venom. Therefore, 1 LD_50_ (13.2 µg) of Ts venom was incubated with the determined minimum neutralizing volume of anti-hyaluronidase serum for 1 h at 37°C. Then, increasing quantities (1.7× increases) of native Ts hyaluronidase were added to the mixture immediately before the turbidimetric assays were performed. Three independent experiments were performed in duplicate.

### *In vivo* neutralization assay

For *in vivo* neutralization tests, samples of Ts venom (1 LD_50_ = 13.2 µg) were incubated for 1 h at 37°C with rabbit pre-immune serum (121.6 µl) or different volumes of anti-hyaluronidase serum (0.94, 3.79, 15.2, 60.8, and 121.6 µl), and then completed with PBS to a final volume of 121.6 µl whenever necessary. After incubation, samples were subcutaneously injected into naive Swiss mice (n = 8 per group). Control animals received only PBS or anti-hyaluronidase serum (n = 4 per group). Within 24 h, the survival time for each mouse was determined, and surviving mice were counted.

To verify the effects of adding native Ts hyaluronidase to neutralized Ts venom on the survival time of mice, Ts venom (1 LD_50_ = 13.2 µg) was incubated for 1 h at 37°C with the minimum neutralizing volume of rabbit anti-hyaluronidase serum (0.94 µl). Subsequently, native Ts hyaluronidase (0.418 µg) was added at the minimum quantity required to recover activity. Minimum quantities of Ts hyaluronidase and anti-hyaluronidase serum were previously determined in the turbidimetric *in vitro* assay. Mice (n = 4) were subcutaneously injected with this mixture of Ts venom (13.2 µg), anti-hyaluronidase serum, and native Ts hyaluronidase. Another group of mice (n = 4) was injected subcutaneously with a mixture of Ts venom (13.2 µg) and native Ts hyaluronidase. Control animals received only hyaluronidase (0.5, 1, 5 µg; n = 4 per group). Animals were observed within 24 h, and the survival time for each mouse was determined.

### Hyaluronidase pharmacological inhibition: *In vivo* assay

For the *in vivo* pharmacological assay, samples of Ts venom (1 LD_50_ = 13.2 µg) were incubated for 1 h at 37°C with aristolochic acid (Sigma-Aldrich), a plant alkaloid nitro compound that inhibits hyaluronidase activity of venom [61]. Multiple venom∶drug (w∶w) ratios were used: 1∶1, 1∶1.25, 1∶1.5, 1∶2, 1∶5, and 1∶10. Samples were diluted in PBS to a final volume of 100 µl. After incubation, samples were subcutaneously injected into naive Swiss mice (n = 8 per group). Control animals received only PBS or only 132 µg of aristolochic acid (n = 4 per group). Animals were observed within 24 h, and surviving mice were counted.

### SPOT peptide immunoassay

The SPOT-synthesis method was performed to determine the pattern of binding of anti-hyaluronidase serum to overlapping peptides from the Ts hyaluronidase isoforms TsHyal-1 or TsHyal-2, thus mapping antigenic peptides in the sequences of both isoforms. It was synthesized peptides corresponding to predicted amino acid sequences of the two Ts hyaluronidase isoforms onto cellulose membranes. Overlapping pentadecapeptides frame-shifted by 3 residues and encompassing sequences from both isoforms were prepared according to the protocol described by Laune *et al.* [62].

Membranes were obtained from Intavis (Koln, Germany). Fmoc amino acids and N-hydroxybenzotriazole were from Novabiochem (Darmstadt, Germany). A ResPep robot (Intavis) was used for the coupling steps. All peptides were acetylated at the N-terminus. After the peptide sequences had been assembled, the side-chain protecting groups were removed by TFA treatment [63].

After an overnight saturation step with blocking buffer (Roche Diagnostics, Penzberg, Germany), membrane-bound peptides were probed with rabbit anti-hyaluronidase serum (1∶2,000). After 90 min of incubation at room temperature, the membranes were washed and incubated with alkaline phosphatase-conjugated anti-rabbit antibodies (Sigma-Aldrich; diluted 1∶4,000) for 60 min at room temperature. Control experiments were performed using rabbit pre-immune serum at the same dilution. After addition of the phosphatase substrate BCIP-MTT (Sigma-Aldrich), peptide spots bound by antibody were detected by their blue coloration. The membrane was placed on a color scanner and scanned without scale reduction. Spot intensities were quantified using ImageJ software (NIH, Bethesda, MD, USA).

### Molecular modeling

The amino acid sequences of TsHyal-1 and TsHyal-2 were input into Blastp (http://blast.ncbi.nlm.nih.gov/Blast.cgi) and the NCBI Protein Data Bank (PDB) to find homologous protein sequences with available three-dimensional (3D) structures. The amino acid sequences of the Ts hyaluronidases were aligned with the identified homologous proteins using Clustal X [64] to identify conserved amino acids. The 3D structures of Ts hyaluronidases were predicted using the structures of Human Hyaluronidase 1 (PDB code: 2PE4; [54]), *Apis mellifera* venom Hyaluronidase (PDB code: 1FCQ; [65]), and the software package Modeller 9.11 [66], [67]. In addition, it was input sequence alignments and predicted structures, including disulfide bonds between residues 172 and 215 for TsHyal-1 and TsHyal-2 (mature predicted proteins), into the modeling program. Models with the lowest folding energy were selected and analyzed with ProSA [68]. The groove volume calculation was performed using the 3V web server (http://3vee.molmovdb.org) [69]. Epitopes mapped by spot-synthesis were located and visualized in the 3D model with PyMol [70].

### Data analysis

Results were expressed as means ± standard error of the mean (S.E.M.). One-way ANOVA with Dunnett's multiple comparison or Bonferroni post-hoc tests were used to analyze the survival times of mice in the *in vivo* assays. The level of significance was set at *p*<0.05. Statistical analysis was performed using GraphPad Prism version 5.0 for Windows (GraphPad Software, San Diego, CA, USA).

## Results

### Hyaluronidase sequence analyses

**Figure 1:**
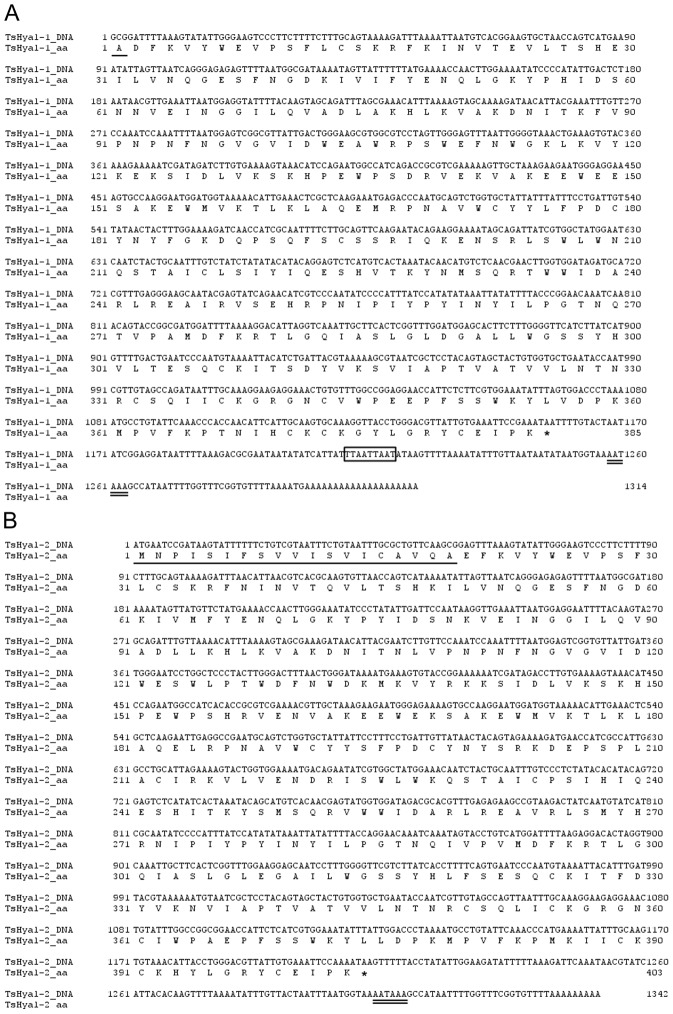
Nucleotide and amino acid sequences of hyaluronidase isoforms from *Tityus serrulatus* venom. cDNA and predicted amino acid sequences of (A) TsHyal-1 and (B) TsHyal-2. Stop codons are marked with asterisks, signal peptide sequences are underlined, and polyadenylation signals are double-underlined. The 9-bp insertion/deletion in TsHyal-1 is boxed.

We obtained the sequences of two isoforms of Ts hyaluronidase, named TsHyal-1 and TsHyal-2. These cDNA sequences have been submitted to the GenBank database under the accession numbers: KF623285 for TsHyal-1 and KF623284 for TsHyal-2. The nucleotide and deduced amino acid sequences of both isoforms are presented in Fig. 1. TsHyal-1 is 1,314 bp and TsHyal-2 is 1,342 bp, and they differ in 136 nucleotides and 1 insertion/deletion of 9 bp. A comparison of both deduced mature amino acid sequences shows 91% similarity and 83% identity with 66 distinct residues. The theoretical mature TsHyal-1 has 384 amino acids, is 44,547 Da, and has a pI of 8.75, whereas the theoretical mature TsHyal-2 has 384 amino acids, is 44,903 Da, and has a pI of 9.17. Both deduced amino acid sequences were classified into family 56 of glycosyl hydrolases by Blastp analysis, as they showed sequence similarities with vertebrate hyaluronidases.

**Figure 2:**
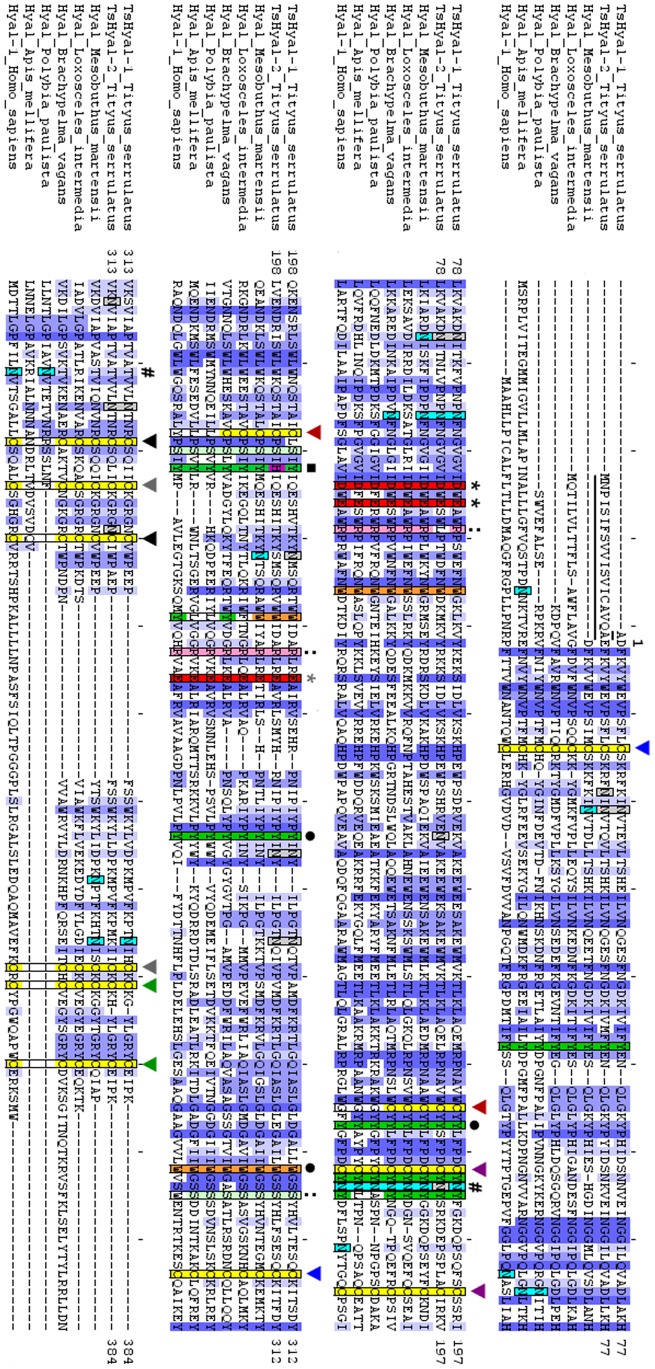
Sequence alignment of TsHyal-1, TsHyal-2, and other hyaluronidases. *Tityus serrulatus* hyaluronidase sequences were aligned with hyaluronidase sequences from *Mesobuthus martensii* (ACY69673), *Loxosceles intermedia* (AGH25912), *Brachypelma vagans* (AFS33217), *Polybia paulista* (ADL09135), *Apis mellifera* (Q08169), and *Homo* sapiens (Q12794). In TsHyal-1 and TsHyal-2 sequences, the signal peptides are underlined, and residue 1 marks the beginning of both mature proteins. Conserved residues are shown in dark blue. The shade of blue indicates the degree of conservation. Conserved cysteine residues are colored yellow, and pairs that form disulfide bonds are denoted with arrows (▾) of matching colors. Residues marked with asterisks (*) represent invariant acidic amino acids. Key catalytic residues in the active site are marked with black asterisks. Other colored residues are involved in proper substrate positioning and enzyme-substrate interactions. Tyrosine and tryptophan residues are colored in green and orange, respectively. Residues denoted with a circle (•) bind to the methyl group of the N-acetyl lateral chain of hyaluronic acid (HA), whereas residues marked with a black square (▪) hydrogen-bond to cleavage site in HA. Arginine and serine residues are colored in pink and light green, respectively. Those marked with two dots (:) are involved in enzyme-substrate interactions. The putative glycosylated asparagines from the literature are marked with light blue squares. Glycosylated asparagines predicted with GPP to TsHyal-1 and TsHyal-2 are marked in grey.

Predicted amino acid sequences of TsHyal-1 and TsHyal-2 were aligned with other known hyaluronidase sequences from diverse organisms (Fig. 2). In pair-wise alignments, TsHyal-1 and TsHyal-2 sequences had, respectively, maximum similarities with hyaluronidases from: scorpion *Mesobuthus martensii* (85% and 80%), spider *Loxosceles intermedia* (65% and 62%), spider *Brachypelma vagans* (66% and 64%), wasp *Polybia paulista* (57% and 57%), honeybee *Apis mellifera* (57% and 58%), and *Homo sapiens* (56% and 57%). Conserved residues are shown in blue (Fig. 2). The shade of blue indicates the degree of conservation, with dark blue indicating a higher degree of conservation.

Critical conserved sites are indicated with different colors; conserved cysteines are yellow and pairs that form disulfide bonds are marked with matching color arrows. We found 12 cysteine residues (forming 6 disulfide bonds) common to all aligned arachnidic hyaluronidases. Aspartic acid (D) and glutamic acid (E) are acidic residues conserved among all the aligned hyaluronidases and are marked in red on Fig. 2. These include the acidic proton (H^+^) donor residues from the active site, D^101^ and E^103^. N-glycosylation sites, predicted by comparison with the other hyaluronidases from the literature, are denoted by light blue squares. Putative N-glycosylation sites in TsHyal-1 and TsHyal-2 deduced by a glycosylation prediction program are denoted by grey squares. Other residues that are also important to correct substrate positioning and substrate-enzyme interaction are shaded in different colors, as described in the Fig. 2 legend.

### Purification of native Ts hyaluronidase

**Figure 3:**
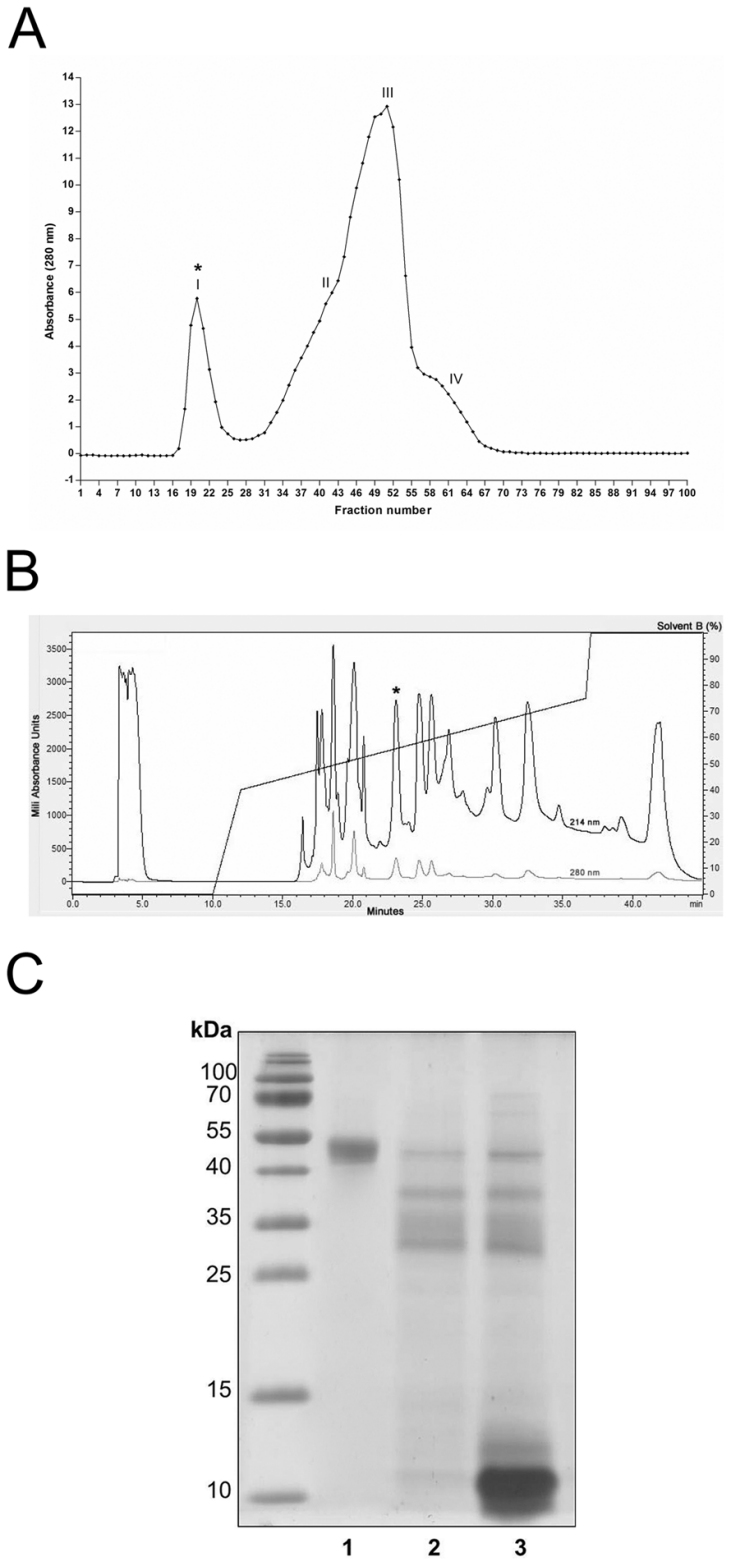
Purification of native hyaluronidase from *Tityus serrulatus* venom. (A) Gel filtration chromatogram of *Tityus serrulatus* venom (225 mg/ml) after fractionation through a Sephadex G-50 Fine column (100 cm×2.5 cm). Samples were eluted with 30% (v/v) acetic acid at a flow rate of 6 ml/h, and elution was monitored at 280 nm. Fractions were assayed for hyaluronidase activity. Only fraction I (marked with an asterisk) was active. (B) Reversed-phase liquid chromatogram of gel filtration fraction I after fractionation through an analytical C8 column (300 Å, 5 µm, 4.6 mm×250 mm). Samples were eluted with a gradient of solution B (0.1% (v/v) TFA in ACN; 0–40% (v/v) B from 10 to 12 min, 40–75% (v/v) B from 12 to 37 min, 75–100% (v/v) B from 37 to 37.5 min, 100% (v/v) B from 37.5 to 45 min) at a flow rate of 1 ml/min, and elution was monitored at 214 and 280 nm. Fractions were assayed for hyaluronidase activity; the asterisk marks the active fraction. (C) 15% (w/v) SDS-PAGE analysis of the hyaluronidase active fraction obtained after reversed-phase chromatography (lane 1; 2 µg), the hyaluronidase active fraction obtained after gel filtration (lane 2; 7 µg), and crude *Tityus serrulatus* venom (lane 3; 12.5 µg). Molecular mass markers are shown on the left.

After the first step of Ts venom fractionation by gel filtration chromatography, the *in vitro* turbidimetric assay showed that only fraction I (Fig. 3A, marked with an asterisk) had hyaluronidase activity (data not shown). Subsequently, fraction I was subjected to RPC, and the resulting fractions were tested for hyaluronidase activity by the *in vitro* turbidimetric assay. Only the fraction eluted between 20 to 25 min (Fig. 3B, marked by an asterisk) had hyaluronidase activity (data not shown). Thus, after two steps of fractionation, Ts native hyaluronidase was purified. Based on the quantity of venom used for purification, we estimated that hyaluronidase comprises 0.38% of crude Ts venom.

Ts venom and the hyaluronidase active fractions derived from the two chromatographic steps were subjected to 15% SDS-PAGE, and gels were stained with Coomassie Blue. Figure 3C shows that there were numerous bands in crude Ts venom and the gel filtration fraction, but only a single band at ∼50 kDa in the reversed-phase fraction. The ∼50 kDa band corresponds to native Ts hyaluronidase, and the presence of only a single band indicates that the purification was successful. MS analysis of hyaluronidase obtained from RPC revealed a compound of 49,312 m/z in the [M+H]^+^ form, which indicated that the molecular mass of the compound was 49.3 kDa (data not shown). MS analysis showed the absence of low molecular weight compounds (5–20 kDa). Also, it was only identified a single compound of approximately 49 kDa, in agreement with hyaluronidase theoretical molecular weight, and its ion fragment (about 24 kDa), which indicated to us that it was a pure sample.

### Anti-hyaluronidase serum production

**Figure 4:**
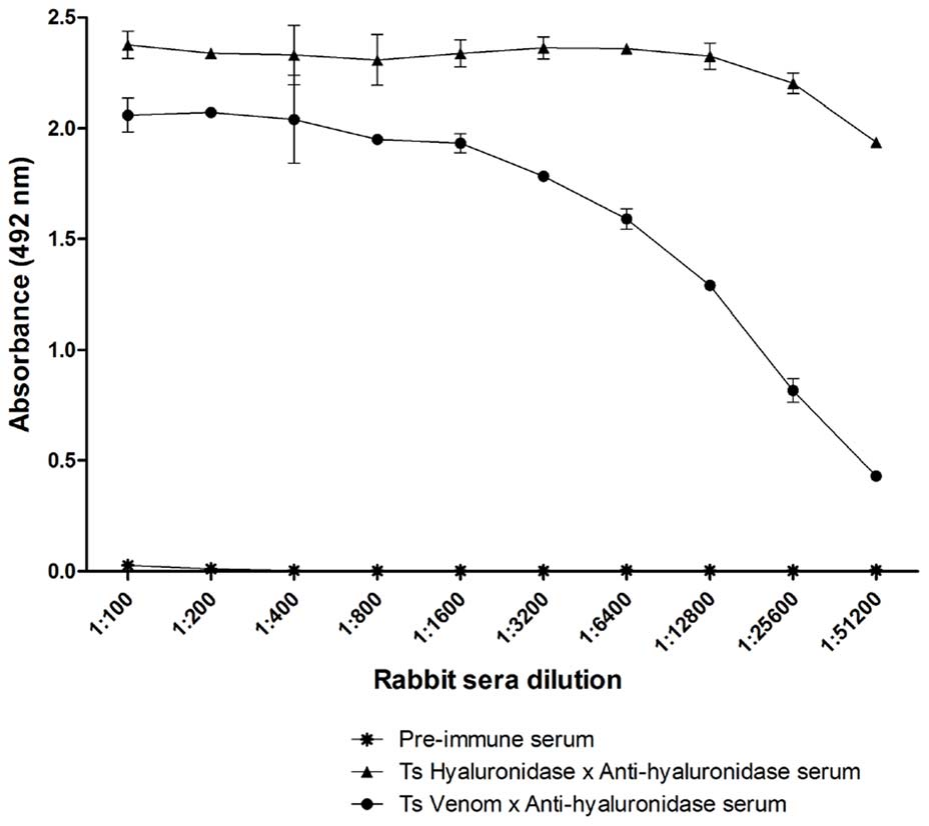
*In vitro* rabbit anti-hyaluronidase serum reactivity. Reactivity of rabbit anti-hyaluronidase serum with native hyaluronidase purified from *Tityus serrulatus* venom (▴) or crude *Tityus serrulatus* venom (•). Pre-immune serum (*) was used as a negative control. ELISA plates were coated with native hyaluronidase purified from *Tityus serrulatus* venom (5 µg/ml) or crude *Tityus serrulatus* venom (5 µg/ml). Rabbit anti-hyaluronidase serum was diluted from 1∶100 to 1∶51,200. A_492_ values shown are the means ± S.E.M. of duplicates.

Purified native Ts hyaluronidase was injected into rabbits to obtain anti-hyaluronidase antibodies. One week after the fourth injection, sera were collected and tested in an indirect ELISA for their reactivity towards native Ts hyaluronidase and crude Ts venom. Anti-hyaluronidase antibodies reacted strongly against purified native Ts hyaluronidase, reaching an A_492_ of 2 with the lowest titer of 1∶51,200 (Fig. 4). Anti-hyaluronidase antibodies also reacted against crude Ts venom, reaching an A_492_ of ∼2 with a titer of 1∶1,600. Pre-immune sera did not react with crude Ts venom or with purified native Ts hyaluronidase (Fig. 4).

### *In vitro* anti-hyaluronidase neutralization assays

**Figure 5:**
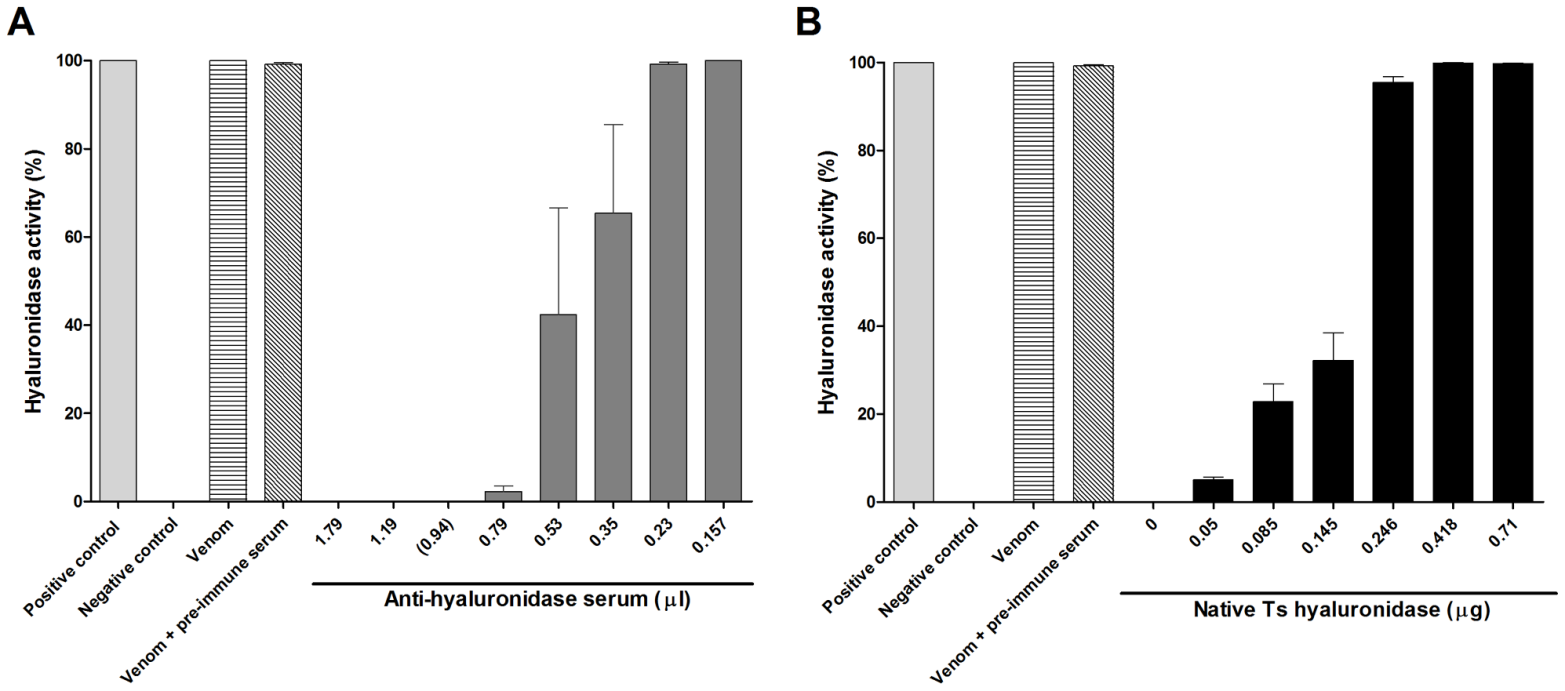
*In vitro* neutralization assays using rabbit anti-hyaluronidase serum. (A) Hyaluronidase activity (%) was measured using the turbidimetric assay. *Tityus serrulatus* venom (1 LD_50_, 13.2 µg) was incubated with different quantities of anti-hyaluronidase serum (0.157–1.79 µl) for 1 h at 37°C. The minimum volume of anti-hyaluronidase serum needed to neutralize *Tityus serrulatus* venom was 0.94 µl (marked with parentheses). (B) Hyaluronidase activity (%) of *Tityus serrulatus* venom (13.2 µg) samples pre-incubated for 1 h at 37°C with anti-hyaluronidase serum (0.94 µl), and subsequently added with increasing quantities of native *Tityus serrulatus* hyaluronidase (0–0.71 µg). Commercial hyaluronidase was used as the positive control. Ultra-pure water served as the negative control. Additional controls were *Tityus serrulatus* venom (13.2 µg) in the absence and presence of pre-immune serum (2 µl). All values are the mean ± S.E.M. of duplicates from 3 independent experiments.

In the turbidimetric assays, commercial hyaluronidase exhibited high hyaluronidase activity, which was referred to as 100% activity (positive control). Ultra-pure water had no enzyme activity, which was referred to as 0% activity (negative control). Ts venom (13.2 µg), in the absence or presence of pre-immune serum (2 µl), presented high enzymatic activity similar to commercial hyaluronidase (positive control) and was considered to be 100% active (Fig. 5AB).

For *in vitro* neutralization assays, we pre-incubated 1 LD_50_ (13.2 µg) of Ts venom with different volumes of rabbit anti-hyaluronidase serum (0.157, 0.23, 0.35, 0.53, 0.79, 1.19, 1.79 µl), and subjected the samples to the turbidimetric hyaluronidase activity assay. First, we observed that the minimum volume of serum needed to neutralize 1 LD_50_ of Ts venom hyaluronidase activity *in vitro* was between 0.79 and 1.19 µl (Fig. 5A). Then, we performed the assay using different quantities of serum between 0.79 and 1.19 µl, and determined that the minimum volume of anti-hyaluronidase serum to neutralize 100% of Ts venom hyaluronidase activity *in vitro* was 0.94 µl (Fig. 5A). Different quantities of purified native Ts hyaluronidase (0–0.71 µg) were added to neutralized Ts venom [1 LD_50_ (13.2 µg) of venom incubated for 1 h at 37°C with 0.94 µl anti-hyaluronidase serum] to recover 100% of the venom's hyaluronidase activity. Fig. 5B shows that 0.418 µg of native Ts hyaluronidase was enough to recover 100% of the hyaluronidase enzymatic activity.

### *In vivo* anti-hyaluronidase neutralization assays

Rabbit anti-hyaluronidase serum effectively neutralized the lethal effects of 1 LD_50_ of Ts venom in mice (Table 1, groups 1–6). Complete (100%) survival was achieved with 121.6 µl of anti-hyaluronidase serum (Table 1, group 6). PBS and anti-hyaluronidase serum controls (Table 1, groups 14 and 15) demonstrated 100% survival of mice, while the 1 LD_50_ Ts venom control (Table 1, group 7) showed 50% survival.

**Table 1:** *In vivo* protection assays.

Group	LD_50_	Test substance	Surviving mice/total mice	Survival percentage (%)
**1**	1	Pre-immune serum (121.6 µl)	4/8	50
**2**	1	Anti-hyaluronidase serum (0.94 µl)	5/8	62.5
**3**	1	Anti-hyaluronidase serum (3.79 µl)	5/8	62.5
**4**	1	Anti-hyaluronidase serum (15.2 µl)	5/8	62.5
**5**	1	Anti-hyaluronidase serum (60.8 µl)	7/8	87.5
**6**	1	Anti-hyaluronidase serum (121.6 µl)	8/8	100
**7**	1	PBS (100 µl)	4/8	50
**8**	1	1∶1 Aristolochic acid (13.2 µg)	4/8	50
**9**	1	1∶1.25 Aristolochic acid (16.5 µg)	5/8	62.5
**10**	1	1∶1.5 Aristolochic acid (19.8 µg)	7/8	87.5
**11**	1	1∶2 Aristolochic acid (26.4 µg)	8/8	100
**12**	1	1∶5 Aristolochic acid (66 µg)	8/8	100
**13**	1	1∶10 Aristolochic acid (132 µg)	8/8	100
**14**	0	PBS (121.6 µl)	4/4	100
**15**	0	Anti-hyaluronidase serum (121.6 µl)	4/4	100
**16**	0	Aristolochic acid (132 µg)	4/4	100

Experimental tests in mice using rabbit anti-hyaluronidase serum or aristolochic acid to neutralize or inhibit *Tityus serrulatus* venom (1 LD_50_, 13.2 µg). Results show the number of surviving mice 24 h after subcutaneous injection of the sample.

**Figure 6:**
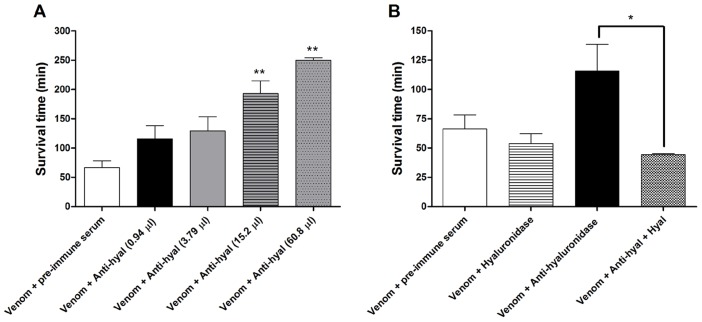
*In vivo* neutralization assays using rabbit anti-hyaluronidase serum. (A) Survival times of mice within 24 h of subcutaneous injection of *Tityus serrulatus* venom (1 LD_50_, 13.2 µg) samples that had been incubated for 1 h at 37°C with pre-immune serum (121.6 µl) or different quantities of anti-hyaluronidase serum (0.94–60.8 µl) were evaluated. ** *p*<0.01 (venom+anti-hyaluronidase group vs. venom+pre-immune serum group). (B) Survival times of mice within 24 h of subcutaneous injection of *Tityus serrulatus* venom (13.2 µg) samples pre-incubated with 0.94 µl of anti-hyaluronidase serum for 1 h at 37°C, with or without the subsequent addition of native *Tityus serrulatus* hyaluronidase (0.418 µg). Mice were also injected with *Tityus serrulatus* venom (13.2 µg) pre-incubated with pre-immune serum (121.6 µl) and *Tityus serrulatus* venom (13.2 µg) supplemented with native hyaluronidase (0.418 µg), and these mice served as controls. * *p*<0.05 (venom+anti-hyaluronidase serum group vs. venom+anti-hyaluronidase serum+native Ts hyaluronidase group). All values are the mean ± S.E.M. of at least 4 experiments.

As little as 15.2 µl and 60.8 µl of anti-hyaluronidase serum delayed death by Ts venom from 66.2±12.0 min to 193±21.6 min and 250.0±4.0 min, respectively, when the serum was incubated with 1 LD_50_ of venom (*p*<0.01; n = 8 per group; Fig. 6A). Furthermore, when native Ts hyaluronidase (0.418 µg) was added to neutralized Ts venom (1 LD_50_ Ts venom+0.94 µl anti-hyaluronidase serum), survival time was reduced from 115.80±22.68 min to 44.35±0.77 min (*p*<0.05; n = 4; Fig. 6B). The injection of increasing doses of hyaluronidase only (from 0.5 to 5 µg) in mice did not cause death (data not shown).

### Aristolochic acid inhibition of hyaluronidase *in vivo*

The hyaluronidase inhibitor aristolochic acid protected mice from death in a dose-dependent manner (Table 1, groups 8–13). Complete (100%) protection of 1 LD_50_ of Ts venom was provided using venom∶drug ratios higher than 1∶2 (13.2 µg venom:26.4 µg aristolochic acid). PBS and aristolochic acid controls (Table 1, groups 14 and 16) showed 100% survival, whereas the 1 LD_50_ Ts venom control (Table 1, group 7) had a 50% survival rate.

### SPOT peptide immunoassay

**Figure 7:**
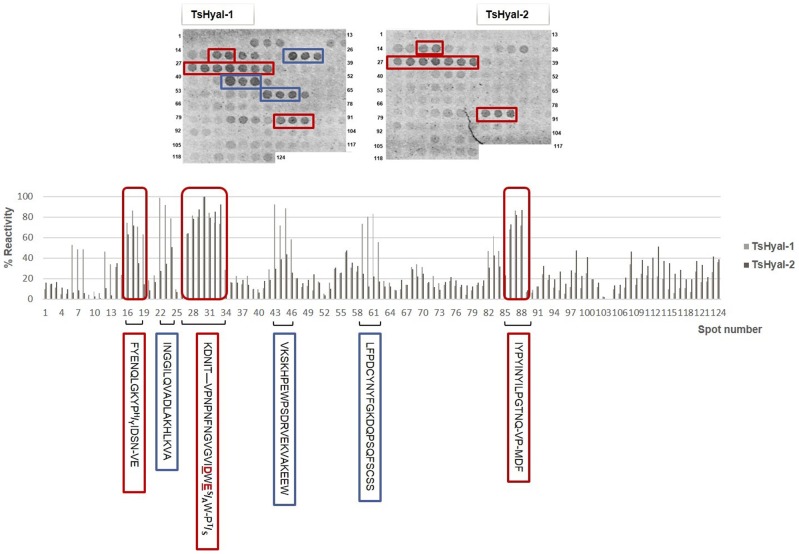
SPOT peptide immunoassay. Reactivity of rabbit anti-hyaluronidase serum to 15-mer overlapping synthetic peptides from the predicted amino acid sequences of TsHyal-1 and TsHyal-2. Peptides were immobilized onto cellulose membranes by the SPOT method, and membranes were probed with a 1∶2,000 dilution of rabbit anti-hyaluronidase serum and a 1∶4,000 dilution of alkaline phosphatase-conjugated anti-rabbit antibodies. Spot intensities were quantified and graphed as percent reactivity against the TsHyal-1 and TsHyal-2 peptide spots. Six antigenic regions with serum reactivities higher than 60% were identified. Three were common to TsHyal-1 and TsHyal-2 (red squares), and three were unique to TsHyal-1 (blue squares). The active site residues D^101^ and E^103^ are marked in red.

Two sets of cellulose-bound synthetic overlapping peptides (15 residues, frame-shifted by 3 residues) that corresponded to amino acid sequences in TsHyal-1 and TsHyal-2 were prepared according to the SPOT-synthesis method. At the end of synthesis, the peptides remained covalently bound to the cellulose membranes and were assayed for antibody reactivity. The binding of anti-hyaluronidase serum with overlapping peptides from TsHyal-1 and TsHyal-2 is shown in Fig. 7. Rabbit anti-hyaluronidase serum reacted differently with some membrane-bound clusters of peptides from each enzyme and recognized more TsHyal-1 peptides than TsHyal-2 peptides. However, the serum clearly bound 3 peptides found in both TsHyal-1 and TsHyal-2 (^46^FYENQLGKYP^H^/_Y_IDSN-VE^63^; ^82^KDNIT-VPNPNFNGfVGVIDWE^S^/_A_W-P^T^/_S_^108^; ^256^IYPYINYILPGTNQ-VP-MDF^276^), as shown in Fig. 7. For this mapping, we only considered serum reactivities higher than 60%.

### Molecular modeling

**Figure 8:**
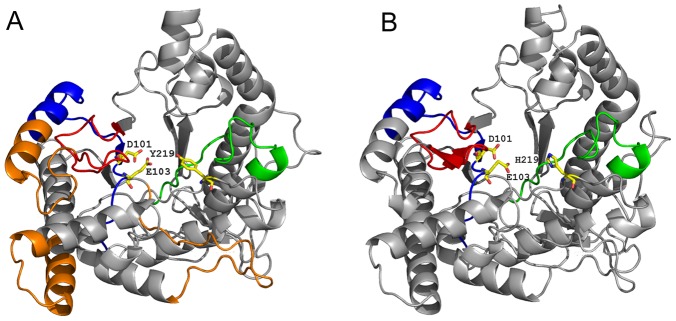
Molecular modeling of TsHyal-1 and TsHyal-2. 3D models for (A) TsHyal-1 and (B) TsHyal-2 were predicted using the crystal structures of Human Hyaluronidase 1 (PDB code: 2PE4) and *Apis mellifera* venom Hyaluronidase (PDB code: 1FCQ) and the software package Modeller 9.11. Identified epitopes were spatially localized to the surfaces of TsHyal-1 and TsHyal-2. Epitopes common to both isoforms are in red, green, and blue, whereas the epitopes found exclusively in TsHyal-1 are marked in orange. Active site residues D^101^ and E^103^ localize to antigenic regions in both isoforms and are represented in the active site groove. An active site amino acid variation exists between both isoforms. TsHyal-1 has tyrosine (Y) at position 219, whereas TsHyal-2 has a histidine (H) at the same position. This difference alters the active site groove slightly and may affect the substrate specificity of each isoform.

We constructed 3D models for TsHyal-1 and TsHyal-2 (Fig. 8). Both isoforms showed the same triosephosphate isomerase (TIM) beta/alpha-barrel fold, but there was an important amino acid variation in the active site groove. TsHyal-1 contains tyrosine (Y) in position 219, whereas TsHyal-2 has a histidine (H) at the same position (Fig. 8A,B). Epitopes identified by the SPOT peptide immunoassay were located on the 3D structures. These epitopes surrounded the active site. Furthermore, even the residues of the enzyme active site, D^101^ and E^103^, were part of the antigenic region (Fig. 8). The 3 epitopes common to both isoforms are shown in blue, red, and green (Fig. 8A,B), whereas the 3 epitopes exclusive to TsHyal-1 are in orange (Fig. 8A).

## Discussion

The major findings of the present work are the description of 2 isoforms of hyaluronidase from Ts venom and the confirmation of the involvement of hyaluronidase in Ts venom lethality, synergistically with neurotoxins. Evolutionarily, hyaluronidases are ubiquitous, are produced by various organisms, including bacteria, insects, arachnids, reptiles, and mammals, and act mainly on the substrates HA and chondroitin [71], [72]. They are involved in many pathophysiological processes, such as embryogenesis, angiogenesis, inflammation, wound healing, and systemic diffusion of venom toxins [45], [73]–[75]. They are also invariant components of snake, bee, scorpion, spider, stonefish, wasp, and lizard venoms [76]–[79]. Nevertheless, few complete amino acid sequences of venom hyaluronidase have been described. Previous to this study, the only available complete hyaluronidase amino acid sequence from scorpion venom was from *Mesobuthus martensii* (ACY69673, predicted sequence). Here, we described the cDNA and predicted amino acid sequences of 2 Ts venom hyaluronidase isoforms, TsHyal-1 and TsHyal-2 (Fig. 1), which had similar molecular characteristics (Fig. 1, 2, 8). The presence of multiple isoforms of hyaluronidase is common in venoms and organisms, including humans. Because hyaluronidases are variable, they are divided into families with distinct characteristics [80], [40], [71].

The predicted amino acid sequences of TsHyal-1 and TsHyal-2 were aligned with the 34 amino acid Ts hyaluronidase sequence obtained by N-terminal Edman degradation (UniProtKB ID: P85841). We found TsHyal-1 to be more identical (97%) to P85841 than TsHyal-2 (85%) (data not shown). Thus, if possible errors in sequencing are considered, TsHyal-1 and P85841 may be the same protein. Amino acid sequences of TsHyal-1 and TsHyal-2 were also aligned with other hyaluronidases, and the conserved residues were primarily involved in protein tertiary structure and enzymatic activity (Fig. 2). Cysteines and disulfide bonds were highly conserved in the catalytic domain; the C^172^-C^215^ disulfide bond (Fig. 2, marked by the red arrow) appeared to be exclusive to arachnidic hyaluronidases and, as Clement *et al.* suggested, may reinforce the stability of the catalytic site [41]. Cysteine residues in the EGF-like domain (from C^331^ to the C-terminus) were also conserved. Conservation of cysteines among hyaluronidases from diverse organisms suggests that they have similar tertiary structures. Also, the predicted 3D structures of TsHyal-1 and TsHyal-2 (Fig. 8) showed that their tertiary conformations were similar.

Crystallographic analysis of *Apis mellifera* hyaluronidase showed that the residues equivalent to TsHyal-1 aspartic acid D^101^ and glutamic acid E^103^ are proton donors that are essential for catalysis [65]. These two amino acids are in the active site grooves of the TsHyal-1 and TsHyal-2 3D models (Fig. 8). Mutations in both of these residues, as well as in E^243^, resulted in loss of enzyme activity [81], [82], [65], [54]. Our alignment showed that these three catalytic residues are conserved in hyaluronidases from multiple organisms (Fig. 2, marked with asterisks). Serine (S) and arginine (R) residues (marked by two dots in the alignment, Fig. 2), have been shown to be important for the enzyme-substrate interaction; the two conserved arginine residues probably guide HA to the active site by electrostatic interactions [65]. The conserved tyrosine (Y, Fig. 2, marked in green) and tryptophan (W, Fig. 2, marked in orange) residues are responsible for correctly positioning and interaction with the substrate in the enzyme active site. The Y and W residues (Fig. 2, marked with black circles) bind to the methyl group of N-acetyl in HA [65], [54], whereas residue Y^219^ (Fig. 2, marked with a black square) binds to the HA cleavage site through hydrogen bonding. It was observed that a mutation in the positioning residue Y^247^ in human Hyal-4 (equivalent to TsHyal-1 Y^219^) altered substrate specificity [83]. Interestingly, our alignment showed that only TsHyal-2 has a histidine (H) instead of a tyrosine (Y) at amino acid 219 (Fig. 2, marked with a black square), and this mutation leads to a slight structural difference in the active site groove of TsHyal-2 when compared to TsHyal-1 (Fig. 8). Thus, even though the structures of TsHyal-1 and TsHyal-2 are similar, TsHyal-1 has a groove volume of 1730 Å^3^, whereas TsHyal-2 has a groove volume of 1987 Å^3^. This variation could cause differences in the substrate specificity between the two isoforms.

To characterize the properties of hyaluronidase, we purified the native enzyme from Ts venom. Native Ts hyaluronidase was first isolated as a 51 kDa protein by Dr. Eliane Arantes' research group (USP, Brazil) using two-step ion-exchange chromatography [47]. In this study, we used gel filtration followed by RPC (Fig. 3) to purify native Ts hyaluronidase. Although the predicted molecular weights of mature TsHyal-1 and TsHyal-2 were 44,547 Da and 44,903 Da, respectively, MS analysis showed that our purified native Ts hyaluronidase was 49.3 kDa. This difference may be a result of unpredicted weight of post-translational modifications, such as N-glycosylation. Feng *et al.* [52] and Jacomini *et al.* [43] also showed similar differences among deduced and experimental hyaluronidases from *Mesobuthus martensii* (scorpion) and *Polybia paulista* (wasp) venoms. As glycosylation is the most common post-translational modification of many eukaryotic intracellular proteins, deglycosylation analysis revealed that N-glycosylation is the most frequent glycosylation type in the hyaluronidase from *M. martensii* venom [52]. Although there are several potential N-glycosylation sites for each sequence aligned, they were not well conserved among each other (Fig. 2, light blue squares) [65], [54], [52], [41], [43]. GPP analysis showed that N-glycosylation sites were not conserved even between TsHyal-1 and TsHyal-2 isoforms. Interestingly, amino acid 323 is an invariant N-glycosylation site found in mammalian hyaluronidases [54]. Further, we observed that N^323^ is conserved among human hyaluronidase, as expected, and wasp hyaluronidases (Fig. 2, marked with a hash tag). The most conserved putative glycosylation site was N^181^ (Fig. 2, marked with a hash tag), but it may not be actually glycosylated, as the *Apis mellifera* hyaluronidase crystal structure showed that site to be buried [65].

In this study, we used native Ts hyaluronidase to generate anti-hyaluronidase serum, which neutralized hyaluronidase activity *in vitro* in a volume-dependent manner. The minimum volume of anti-hyaluronidase serum that effectively neutralized 1 LD_50_ of Ts venom *in vitro* was 0.94 µl (Fig. 5A). Despite it is such a small amount of serum, we believe it was sufficient to completely neutralize the enzyme activity from 1 LD_50_ of venom, because native hyaluronidase constitutes only 0.38% of whole Ts venom. Similarly, the methodology used by Pessini *et al.* [47] recovered 0.35% of hyaluronidase from Ts whole venom. *In vivo* neutralization assays showed that neutralized venom (1 LD_50_ of venom+121.6 µl of anti-hyaluronidase serum) increased the survival time and inhibited death of mice (Table 1). The delay in death that resulted from neutralization of Ts venom was observed in a serum volume-dependent way (Fig. 6A). This volume dependence illustrates the efficacy of this serum to neutralize venom toxicity. Nevertheless, in our assays, anti-hyaluronidase serum, even in high volumes, was not able to neutralize 2 LD_50_ of Ts venom in mice (data not shown). Similarly, Girish and Kemparaju [84] produced a rabbit serum against native hyaluronidase from *Naja naja* snake venom, called anti-NNH1, that neutralized the hyaluronidase activity of the snake venom *in vitro*. Additionally, *in vivo* neutralization assays showed that mice injected with crude snake venom pre-incubated with anti-NNH1 serum presented more than a two-fold increase in survival time when compared to mice injected with venom alone.

When 1 LD_50_ of Ts venom was neutralized with anti-hyaluronidase serum (0.94 µl), the quantity of native hyaluronidase capable of recovering 100% of the neutralized hyaluronidase activity *in vitro* was 0.418 µg (Fig. 5B). *In vitro*, we observed that an excess of serum managed to neutralize the hyaluronidase activity of Ts venom and the activity of the native hyaluronidase added to the sample (data not shown). In that sense, *in vivo* neutralizing assay was carried out incubating 1 LD_50_ of Ts venom with only 0.94 µl of serum. Then, 0.418 µg of native Ts hyaluronidase was added to the incubated neutralized venom and the mixture was injected in mice. Interestingly, we showed that the addition of Ts native hyaluronidase significantly decreased the survival time of mice, in comparison to the group injected with neutralized Ts venom alone (Fig. 6B). In contrast, when native Ts hyaluronidase was added to 1 LD_50_ of Ts venom without any serum, the mouse survival time did not differ from the control group injected with 1 LD_50_ of Ts venom and pre-immune serum (Fig. 6B). We believe that the hyaluronidase in Ts venom is sufficiently active so that the addition of purified native Ts hyaluronidase is inconsequential. Thus, extra doses of this enzyme would not interfere with envenomation. Also, the administration of native Ts hyaluronidase alone (maximum of 5 µg) was not toxic to mice (data not shown).

Western blot analysis showed that rabbit anti-hyaluronidase serum reacted with high-molecular-weight proteins (>25 kDa) other than hyaluronidase (data not shown). Scorpion venom proteins within this weight range most likely correspond to enzymes, such as metalloproteases, that may also facilitate the spread of venom by degrading extracellular matrix proteins, including laminin and type IV collagen [35], [17], [36]. To confirm that the neutralization effects observed in our *in vivo* assays (Fig. 6; Table 1) were a result of the inhibition of hyaluronidase, we used a pharmacological hyaluronidase inhibitor.

We used aristolochic acid, an alkaloid isolated from the Indian medicinal plant *Aristolochia indica* [85]. This compound has been well characterized, mainly in snake venoms, as a hyaluronidase inhibitor [61], [84], [86]. Here, we showed that aristolochic acid protected mice from death in a dose-dependent manner when injected with 1 LD_50_ of Ts venom (Table 1). Aristolochic acid inhibited *in vitro* Ts hyaluronidase activity (data not shown). Aristolochic acid has been shown to inhibit phospholipase A_2_ [87]–[90]; however, phospholipase A_2_ activity was not found in Ts venom [36]. Thus, we conclude that aristolochic acid primarily inhibits hyaluronidase in Ts venom. This finding confirms that the *in vivo* neutralization effects of anti-hyaluronidase serum were a result of hyaluronidase inhibition.

Although snake venom hyaluronidases have been recognized as venom “spreading factors” for many years, they are among the least-studied components of snake venoms [79]. Hyaluronidase promotes venom dissemination, thereby increasing its local and systemic effects. It has also been demonstrated to potentiate the effects of hemorrhagic toxins from snake venoms [37], [59], [45], [73]. Therefore, many neutralization and inhibition studies have been performed on snake venom hyaluronidase using specific antiserum or pharmacological inhibitors. These neutralization and inhibition strategies reduced the local and systemic effects of envenomation and prolonged the survival time of animals injected with snake venoms [91], [92], [44], [84]. We observed similar neutralization and inhibition of *T. serrulatus* scorpion venom with anti-hyaluronidase serum and the pharmacological inhibitor aristolochic acid.

In the last part of this study, we determined which epitopes of TsHyal-1 and TsHyal-2 were recognized by protective anti-hyaluronidase serum using the SPOT peptide immunoassay. Systematic mapping of continuous epitopes recognized by anti-hyaluronidase serum found 3 antigenic regions common to both TsHyal-1 and TsHyal-2 (Fig. 7, marked in red). Among these regions, the second one (equivalent to ^82^KDNITKFVPNPNFNGVGVIDWEAWRPS^108^ in TsHyal-1) was more conserved between the aligned hyaluronidase sequences (Fig. 2). This region contains the active site residues D^101^ and E^103^. The recognition of TsHyal-1 and TsHyal-2 epitopes was poorly achieved by a commercially available polyvalent antiscorpion venom serum, however, this serum was able to neutralize Ts venom hyaluronidase activity *in vitro* (data not shown). The 3 antigenic regions identified by SPOT analysis were mapped onto the 3D models of TsHyal-1 and TsHyal-2, and were found to surround the active sites (Fig. 8). This result indicates that the neutralization of Ts venom by anti-hyaluronidase serum observed in our *in vitro* and *in vivo* assays was a result of the binding of serum antibodies to specific residues in the Ts hyaluronidase active site. Thus, this study provides a plausible hypothesis to explain the molecular bases of the neutralizing activity of anti-hyaluronidase serum antibodies. However, antibodies that bind to conformational epitopes were not considered by this technique. Either way, the discovery of relevant hyaluronidase epitopes represents an important achievement to specific antivenom assembly. Synthetic peptides that mimic the region found to be dominantly antigenic in the hyaluronidase structure might be useful as supplementary immunogens to be used in the antivenom production, raising the concentration of specific anti-hyaluronidase antibodies in the final product, therefore improving the effectiveness of the venom neutralization.

Given all the results, we could hypothesize the applicability of hyaluronidase neutralization and inhibition to scorpionism treatments. Because hyaluronidase facilitates initial venom dissemination, its activity must be blocked immediately after envenomation. It would be interesting to use anti-hyaluronidase serum as a complement to antivenom therapy for cases in which treatment can be administered directly after the accident. Otherwise it would be a late and pointless strategy. Hyaluronidase inhibition by drugs or natural extracts has also been suggested as a first-aid treatment for envenomation [45], [86]. Pharmacological hyaluronidase inhibition would extend the time available between a bite and antivenom administration, and may reduce the antivenom dose required for neutralization [79]. Here, the anti-hyaluronidase serum produced was functional as an investigation tool of the pharmacological role of hyaluronidase in the envenomation by *T. serrulatus*.

In this study, anti-hyaluronidase serum was found to effectively neutralize Ts venom toxicity and to delay the death of envenomed mice. This finding demonstrates that hyaluronidase plays a critical role in Ts systemic toxicity. Thus, our findings challenge the notion that only neurotoxins are relevant to Ts envenomation.
